# The DARC-null trait is associated with moderate modulation of NK cell profiles and unaltered cytolytic T cell profiles in black South Africans

**DOI:** 10.1371/journal.pone.0242448

**Published:** 2020-11-19

**Authors:** Kewreshini K. Naidoo, Zesuliwe B. Shangase, Tabassum Rashid, Ayanda Ngubane, Nasreen Ismail, Thumbi Ndung’u, Christina F. Thobakgale

**Affiliations:** 1 HIV Pathogenesis Programme, Doris Duke Medical Research Institute, University of KwaZulu-Natal, Durban, South Africa; 2 Ragon Institute of MGH, MIT and Harvard University, Cambridge, MA, United States of America; 3 Africa Health Research Institute, Durban, South Africa; 4 Max Planck Institute for Infection Biology, Berlin, Germany; 5 Division of Infection and Immunity, University College London, London, United Kingdom; 6 Faculty of Health Sciences, Centre for HIV and STIs, National Institute for Communicable Diseases, University of the Witwatersrand, Johannesburg, South Africa; Boston University, UNITED STATES

## Abstract

The Duffy Antigen Receptor for Chemokines (DARC)-null trait, common among persons of African descent and associated with lower absolute neutrophil counts (ANCs), may be linked to increased risk to certain infections including HIV-1 but the underlying causes are poorly understood. We hypothesized that DARC-null-linked neutropenia may negatively impact neutrophil immunoregulatory modulation of other immune cells such as natural killer (NK) and CD8+ T cells leading to altered phenotype, functionality and homeostatic activity of these immune cells. HIV-1 uninfected (n = 20) and HIV-1 chronically infected (n = 19) participants were assessed using multi-parametric flow cytometry to determine NK and CD8+ T cell counts, phenotypic profiles, and cytokine production and degranulation. Annexin V and carboxyfluorescein succinimidyl ester (CFSE) staining were used to examine NK cell survival and NK cell and CD8+ T cell proliferation respectively. Participants were genotyped for the DARC-null polymorphism using allelic discrimination assays and ANCs were measured by full blood count. In HIV uninfected individuals, a reduction of total NK cell counts was noted in the absence of DARC and this correlated with lower ANCs. HIV uninfected DARC-null subjects displayed a less mature NK cell phenotype. However, this did not translate to differences in NK cell activation or effector functionality by DARC state. Whilst HIV-1 infected subjects displayed NK cell profiling that is typical of HIV infection, no differences were noted upon DARC stratification. Similarly, CD8+ T cells from HIV infected individuals displayed phenotypic and functional modulation that is characteristic of HIV infection, but profiling was unaffected by the DARC-null variant irrespective of HIV status. Overall, the data suggests that the DARC-null polymorphism and lower ANCs does not impede downstream cytolytic cell priming and functionality.

## Introduction

The Duffy antigen receptor for chemokines (DARC), encoded by the DARC gene located on chromosome 1 (q21-q25), is a glycosylated membrane receptor expressed primarily on erythrocytes, and to some extent on vascular endothelial and neuronal cells. The receptor is multi-specific and binds several pro-inflammatory chemokines hence acting as a chemokine “sink” and regulating plasma chemokine levels. Clinically, expression of DARC on erythrocytes has been implicated due to the dependency of *Plasmodium vivax* to enter erythrocytes via DARC and establish malarial infection. A single nucleotide substitution (-46T>C) within the DARC promoter region results in selective loss of DARC expression on erythrocytes only; and thus confers relative resistance to certain malarial parasites. The DARC-null phenotype is common amongst African ancestry populations and a plausible consequence of positive selection pressure in regions where *P*. *vivax* has historically been endemic [1–3].

Admixture mapping identifies the DARC-null variant as the principal determinant of benign ethnic neutropenia [4, 5] which occurs in 25–50% of persons of African ethnicity [6]. Although the molecular mechanisms for this association remain poorly understood it is postulated that DARC expression is involved in the regulation of peripheral chemokine levels that affect neutrophil chemotaxis, migration and localization [7]. The DARC-null phenotype has been linked to several health issues including asthma, rheumatoid arthritis, cancer and transplant rejection [8]. Furthermore, DARC-null neutropenia has been associated with an increased susceptibility to HIV-1 acquisition [9, 10] and an increased risk of mother-to-child HIV transmission [11]. The effect of the DARC-null state on disease progression is less certain, with studies on the impact of the DARC-null trait on HIV disease progression and survival advantage yielding contrasting results [12–15].

Although DARC-null neutropenia has been implicated in HIV-1 acquisition and disease progression, the underlying causes for these possible associations remain unclear. We recently reported that neutrophil effector mechanisms were unimpaired in individuals with the DARC-null trait irrespective of HIV status [16]. Apart from their effector functions, neutrophils form regulatory communication networks with several immune cellular subsets [17, 18]. This was particularly evident in studies which demonstrated the essential role of neutrophils in the mediation of NK cell maturation [19–21] and the ability of antigen-pulsed neutrophils to induce cytotoxic T cell differentiation in the absence of functional major histocompatibility complex (MHC) class I presenting cells [22]. NK cells and cytotoxic T cells represent cytolytic lymphocytes, a distinct class of potent effectors of cell-mediated immunity. The significance of cytolytic lymphocytes have been highlighted in the early containment and control of HIV-1 infection [23, 24] and is supported by the evolution of viral strategies to evade cytolytic immune mechanisms [25].

Thobakgale and Ndung’u (2014) recently hypothesized that the low circulatory levels of neutrophils present in DARC-null individuals could impact both the priming and functional capacity of cytolytic subsets, leading to an impaired ability to mediate an effective response during HIV-1 infection. In this study we addressed this hypothesis by assessing the impact of the DARC-null variant and lower neutrophil counts on the cellular phenotypic patterns and functionality of NK cells and CD8+ cytotoxic T cells in individuals from an HIV prevalent region in South Africa. We found that in HIV negative individuals, absence of the DARC genotype was associated with lower NK cell numbers and higher frequencies of less differentiated NK cells. However, this immature phenotype did not amount to functional differences when NK cells were assessed by DARC genotype. In contrast, no differences were exhibited in the profiles of NK cells or CD8+ T cells of HIV-1 infected individuals upon stratification by DARC genotype.

## Materials and methods

### Participant recruitment

HIV negative participants were recruited from the Females Rising through Education, Support and Health (FRESH) cohort, an acute HIV infection monitoring cohort that targets high risk women. HIV-1 subtype C chronically infected, treatment naïve subjects were recruited from the HIV Pathogenesis Programme (HPP) Acute Infection Cohort, an acute to chronic HIV infection cohort based at the Prince Mshiyeni Memorial Hospital. Both cohorts are located in the Umlazi Township in Durban, KwaZulu-Natal (KZN), South Africa. Both studies attained ethical approval from the University of KwaZulu-Natal (UKZN) Biomedical Research Ethical Committee (BREC) (FRESH BREC Reference Number: BF131/11 and HPP Acute Infection Cohort BREC Reference Number: E036/06). All participants gave written informed consent in accordance with the Declaration of Helsinki. This particular study obtained further ethical clearance as a subsidiary of the above mentioned studies (BREC Reference Number: BE229/15). A total of 39 individuals sub-divided into HIV negative donors (n = 20) and HIV-1 chronically infected individuals (n = 19) were assessed. These individuals were further stratified by DARC status.

### ANCs, CD4 counts and viral load

Blood for full blood counts, CD4+ T cell count measurement and viral load quantification was collected in Ethylenediaminetetraacetic acid (EDTA) anticoagulated vacutainer tubes (Becton Dickinson (BD), Franklin Lakes, New Jersey, USA). ANCs were enumerated by full blood count using the automated XN 1000 Haematology Analyser (Sysmex, Kobe, Hyōgo, Japan). CD4 counts were measured using BD Trucount and analysed on a four-parameter FACS Calibur flow cytometer (BD). Viral loads were determined using the NucliSENS EasyQ HIV1 v2.0 kit with a detection limit of 20 copies/ml (BioMérieux, Marcy-l'Étoile, France).

### Quantification of antiretroviral therapy (ART) drugs in plasma

This study aimed to recruit HIV-1 chronically infected, treatment naïve individuals. ART usage in chronically infected patients was self-reported. Certain individuals maintained viral loads below 1000 RNA copies/ml at the time of assessment. To rule out ART drug use, plasma samples were collected from these study participants and screened for ART drugs using a quantitative liquid chromatography coupled with tandem mass spectrometry (LC-MS/MS) method. The method screened for nine ART drugs commonly used for HIV-1 clinical management in South Africa, namely Emtracitabine, Tenofovir, Lopinavir, Ritonavir, Nevirapine, Abacavir, Lamivudine, Zidovudine and Efavirenz. A plasma sample volume of 50 μl was processed using a protein precipitation method, ART drug analytes were chromatographically separated on a Agilent Zorbax Eclipse Plus C18 (2.1 x 50 mm, 3.5 μm) HPLC column (Agilent Technologies, Santa Clara, California, USA), detected using an AB Sciex 5500 triple quadrupole mass spectrometer (Sciex, Framingham, Massachusetts, USA) and quantitated using Analyst® 1.6.2 software (Sciex).

### DARC genotyping

DARC -46T → C (rs2814778) single-nucleotide polymorphism (SNP) genotyping was performed by TaqMan allelic discrimination assays which has been previously verified by direct sequence analysis [13]. Briefly, genomic DNA was isolated from stored buffy coats using the QIAamp DNA Blood Midi kit (Qiagen, Hilden, Germany) according to manufacturer’s instructions. DNA concentration was standardised at 50 ng/μl with PCR grade water. A cocktail containing TaqMan Genotyping master mix (Life Technologies, Carlsbad, California, USA) and predesigned probes for the DARC gene (SNP ID: rs2814778, Applied Biosystems, Foster City, California, USA) was used to amplify target sequence in 50 ng genomic DNA by real time PCR in the LightCycler 480 (Roche, Basel, Switzerland) according to the manufacturer’s protocol.

### PBMC isolation, cryopreservation and thawing

Blood collected in sodium heparin BD Vacutainers (BD) from each participant was processed within 6 hours of collection. Whole blood was used for lymphocyte fraction preparation by density centrifugation on a Histopaque-1119 cushion (Sigma-Aldrich, St. Louis, Missouri, USA) at 800 x g for 20 minutes, low brakes as previously described [16, 26]. Cell counts were determined by 1:5 dilution with Trypan Blue Stain (Gibco) using a haemocytometer under a light microscope. Cells were prepared for cryopreservation by refrigerated centrifugation and re-suspended at a final concentration of 10 million cells/ml in ice cold freezing solution containing 1:10 Dimethyl Sulfoxide (Merck Millipore, Billerica, Massachusetts, USA) and Foetal Bovine Serum (FBS, Gibco). Samples were stored a liquid nitrogen Biorack ultralow freezer (-160°C to -196°C) for long term storage and subsequently batched for phenotypic and intracellular staining (ICS) assays.

Cryopreserved cells were rapidly thawed, washed twice in pre-warmed R10 medium (RPMI Medium 1640 (Gibco) containing 10% gamma irradiated, heat inactivated FBS, 1% L-Glutamine, 1% Penicillin Streptomycin and 1% Hepes buffer 1M (all from Lonza, Basel, Basel-Stadt, Switzerland) at 500 x g for 8 minutes at room temperature. Cells were rested in R10 medium in a 37°C, 5% CO_2_ incubator for at least 2 hours. Sample viability and cell counts were determined by trypan blue exclusion, and cells were re-suspended at 10 million cells/ml in R10 medium for subsequent assays. Cell viability was not statistically different by HIV or DARC status. Median viability was 92% in HIV negative and 88% in HIV infected individuals (p = 0.12). By DARC status, median viability in HIV negative individuals was 91% and 94% in DARC-null and DARC-positive individuals respectively (p = 0.82), and in HIV infected individuals median cell viability was 88% and 84% in DARC-null and DARC-positive individuals respectively (p = 0.59).

### PBMC *ex vivo* phenotyping and *in vitro* ICS staining

All peripheral blood mononuclear cell (PBMC) staining was performed in 96-well plates. Each staining / fixation incubation was for 20 minutes at room temperature in the dark, followed by washing with Dulbecco's phosphate-buffered saline (dPBS) and centrifuged at 850 x g for 6 minutes. For phenotype staining, one million cells were stained with LIVE/DEAD Fixable Aqua Dead Cell Stain (Invitrogen, Carlsbad, California, USA) to exclude dead cells, followed by cell surface staining with the following monoclonal antibodies: anti-CD3 Phycoerythrin (PE)-CF594 (clone UCHT1), anti-CD14 V500 (clone M5E2), anti-CD19 V500 (clone HIB19) (to exclude of T-cells, monocytes and B cells respectively), NK cell markers Alexa-Fluor-700 conjugated anti-CD56 (clone B159) and anti-CD16 Allophycocyanin (APC)-Cy7 (clone 3G8) and cytolytic T cell marker PE-Cy7 conjugated anti-CD8 (clone SK1, all from BD Biosciences). In addition, to assess activation and differentiation, cells were stained with anti-CD69 fluorescein isothiocyanate (FITC, clone FN50 BD Biosciences), anti-CD38 PE (clone HB7 BD Biosciences), anti-HLA-DR Brilliant Violet (BV) 711 (clone L243 BioLegend, San Diego, California, USA), anti-PD-1 BV421 (clone EH12.2H7 BioLegend) and anti-CD57 APC (clone NK-1 BD Biosciences), before addition of fixation Medium A (Invitrogen).

For *in vitro* functional assays: 1 million cells were assessed for cytokine production and degranulation capacity in the presence of degranulation marker anti-CD107a PE-Cy5 (clone H4A3 BD Biosciences), BD GolgiStop protein transport inhibitor (BD Biosciences) and Brefaldin A (Sigma-Aldrich) at a final concentration of 1 μg / 1x10^6^ cells. For NK cell assays: PMBCs were further stimulated with either R10 medium alone, a cocktail containing Recombinant Human Interleukin (IL)-2, Recombinant Human IL-15 (both from R&D Systems, Minneapolis, Minnesota, USA), and Recombinant Human IL-18 (Medical & Biological Laboratories, Japan) all at a final concentration of 10 ng / 1x10^6^ cells alone, K-562 cell line alone (ATCC, Manassas, Virginia, USA; Cat # ATCC® CCL-243™) at an effector:target ratio of 10:1, or both for 18 hours at 37°C, 5% CO_2_. For CD8+ T cell assays: PBMCs were further stimulated with either R10 medium alone, Gag or Env peptide pools (gp41 and gp120) all at a final concentration of 2 μg / 1x10^6^ cells. Cells stimulated with either 1 μg Staphylococcus enterotoxin B (SEB) per 1x10^6^ cells or 1 μg Phorbol-Myristate-Acetate (PMA) and 0.5 μg ionomycin per 1x10^6^ cells served as positive controls. Cells were incubated for 16 hours at 37°C, 5% CO_2_. Following overnight culturing, samples were stained with LIVE/DEAD Fixable Aqua Dead Cell Stain followed by surface staining with anti-CD3 PE-CF594, anti-CD14 BV650 (clone M5E2), anti-CD19 BV650 (clone HIB19, both from BioLegend), and either Alexa-Fluor-700 conjugated anti-CD56 and anti-CD16 APC-Cy7 for NK cell assessment or anti-CD8 Alexa-Fluor-700 (clone RPA-T8 BD Biosciences) and anti-CD4 APC-Cy7 (clone RPA-T4 BioLegend) for cytolytic T cell assessment. For NK cell assessment, samples were also stained with anti-CD158a PE (clone HP-3E4), anti-CD158b PE (clone CH-L, both from BD Biosciences), anti-CD158e1/e2 PE (clone Z27.3.7) and anti-NKG2A APC (clone Z199, both from Beckman Coulter, Brea, California, USA). This was followed by fixation with Medium A and ICS staining with antibody cocktail containing cytokine markers anti-Tumor necrosis factor (TNF)-α PerCP-Cy5.5 (clone Mab11) and anti-Interferon gamma (IFN-γ) PE-Cy7 (clone B27, both from BioLegend) in the presence of permeabilisation buffer Medium B (Invitrogen). Details of antibodies are summarized in S1 Table.

### Survival assay

NK cells were assessed for survival by plating one million PBMCs in 96-well plates with either R10 medium alone or in the presence of K-562 cell line (effector:target ratio of 10:1) and 10ng each of Recombinant Human IL-2, Recombinant Human IL-15 and Recombinant Human IL-18 and incubated for 18 hours at 37°C, 5% CO_2_. Following overnight culture, cells were stained for surface markers with antibody cocktail containing anti-CD3 APC (clone UCHT1), anti-CD56 Alexa-Fluor-700, anti-CD16 APC-Cy7, anti-CD14 V500 and anti-CD19 V500. FITC conjugated Annexin V and propidium iodide (PI) staining solution were included in the antibody cocktail in the presence of Annexin V binding buffer (all from BD Biosciences) to detect for cell death via apoptosis and necrosis respectively. Cells were stained for 20 minutes at room temperature in the dark, washed in 2% FCS in dPBS and centrifuged at 850 x g for 6 minutes, followed by fixation with Medium A (Invitrogen). Details of antibodies are summarized in S1 Table.

### Proliferative capacity

To assess the replicative capacity of NK cells and CD8+ T cells, PBMCs were washed once with dPBS to remove traces of FCS and centrifuged at 500 x g for 6 minutes. Pelleted PBMCs were stained with carboxyfluorescein succinimidyl ester (CFSE) dye (Invitrogen) for 7 minutes at 37°C with 5% CO_2_. Cells were immediately washed with ice cold FCS to stop the reaction and centrifuged at 500 x g for 6 minutes, washed once with R10 medium, centrifuged at 500 x g for 6 minutes and resuspended at 10 million cells/ml in R10 medium. For NK cells: One million PBMCs were plated in 96-well plates with either R10 medium alone or in the presence of 10 ng each of Recombinant Human IL-2, Recombinant Human IL-15 and Recombinant Human IL-18, and incubated for 5 days at 37°C with 5% CO_2_. On day 3, 100 μl R10 supernatant was removed from each well without disturbing the cell pellet and replenished with fresh R10 medium. For CD8+ T cells: Half a million cells were stimulated with either gag peptide at 2 μg / 1x10^6^ cells or phytohemagglutinin (PHA) at a final concentration of 10 μg / 1x10^6^ cells. Unstimulated cells (R10 medium only) served as a negative control. Cells were cultured at 37°C with 5% CO_2_ for 7 days. On day 4, 100 μl R10 supernatant was removed from each well without disturbing the cell pellet and replenished with fresh R10 medium. Following incubation, cells were stained with LIVE/DEAD Fixable Aqua Dead Cell Stain to exclude dead cells, followed by cell surface staining with monoclonal antibodies: anti-CD3 APC, anti-CD14 BV650, anti-CD19 BV650, and either Alexa-Fluor-700 conjugated anti-CD56 and anti-CD16 APC-Cy7 for NK cell assessment or anti-CD8 Alexa-Fluor-700 and anti-CD4 APC-Cy7 for cytolytic T cell assessment, before fixing with Medium A. Details of antibodies are summarized in S1 Table.

### Quantification of plasma cytokines / chemokines

The Bio-Plex Pro™ Human Cytokine 17-plex Immunoassay (Bio-Rad, Hercules, California, USA) was used to analyze plasma IL-1β, IL-2, IL-4, IL-5, IL-6, IL-7, IL-8, IL-10, IL-12 (p70), IL-13, IL-17A, granulocyte colony stimulating factor (G-CSF), granulocyte macrophage colony stimulating factor (GM-CSF), monocyte chemoattractant protein-1 (MCP-1), macrophage inflammatory protein (MIP)-1β, TNF-α and IFN-γ in duplicate according to manufacturer’s protocol and read on the Bio-Plex™ 200 system (Bio-Rad, USA).

### Sample acquisition and statistical analysis

Samples were acquired on an LSR Fortessa or LSRII flow cytometer (BD). Daily routine instrument QC was performed using Cytometer Setup and Tracking beads (BD Biosciences). Compensation was performed for each experiment using the Anti-Mouse Ig, κ/Negative Control Compensation Particles Set (BD Biosciences). Compensation was calculated using FACS Diva Software (BD) on the experiment template used to acquire experimental samples. At least 250,000 events were recorded per experimental sample. Fluorescence minus one (FMOs) were used in each experiment to exclude background fluorescence in the gating strategies for each activation/differentiation marker. For ICS assays, degranulation and cytokine frequencies were examined after background subtraction from unstimulated controls. For survival assays, FMOs for Annexin V and PI were prepared with each experiment to exclude background fluorescence in the gating strategies. For proliferation assays, unstimulated cells were used to subtract background from the stimulated cells for each patient sample. FlowJo Software Version 9 (TreeStar, Inc., Ashland, Oregon, USA) was used for sample analysis. Statistical comparisons between studied groups was examined with two-tailed Mann-Whitney U tests with an alpha value of 0.05 using GraphPad Prism Version 5 software (GraphPad software Inc., La Jolla, California, USA). Data are expressed as medians with interquartile range. Low NK cell frequencies in some individuals made downstream gating of certain phenotypic and functional data unreliable and this data was omitted.

## Results

This study aimed to evaluate the phenotypic characteristics and functional responses of cytolytic immune cells in the presence of the DARC-null variant and lower ANCs. We recently described neutrophil effector functions in these individuals [16]. Here we conducted further analyses to examine NK cell and CD8+ T cell profiles in 20 HIV-uninfected and 19 HIV-1 chronically infected Zulu / Xhosa black South Africans. The clinical data for these individuals are presented in Table 1. All HIV negative participants were females, while 95% of the HIV-1 infected group were females. Twenty two of thirty nine subjects (56%) were found to possess the DARC-null trait. There were no differences in median age by HIV status (21 and 23 years for HIV negative and HIV-1 infected participants respectively) or DARC trait (21 years for DARC-null and 22 years for DARC-positive in HIV negative individuals; 23 years for both DARC-null and DARC-positive in HIV positive individuals). Absolute CD3 counts did not differ by HIV status (p = 0.79) or DARC variant (p = 0.97 in HIV negative individuals and p = 0.24 in HIV positive individuals). Although absolute CD4 counts were significantly lower in HIV-1 infected compared to HIV negative individuals (p = 0.0025), assessment by DARC status showed no differences in CD4 counts (p = 0.70 in HIV negative individuals and p = 0.44 in HIV positive individuals). Furthermore, no significant differences were observed in viral loads by DARC genotype in HIV infected individuals (p = 0.90) all of whom were antiretroviral therapy naïve (Table 1). Total white blood cells were comparable between HIV negative and HIV positive individuals (p = 0.11); but were significantly lower in DARC-null persons (p = 0.0061 in HIV negative individuals and p = 0.0004 in HIV positive individuals) and positively correlated with ANCs irrespective of HIV status (p<0.0001, S2 Table). Similarly, ANCs did not differ significantly by HIV status (p = 0.08). However, DARC stratification indicated significantly lower ANCs in DARC-null compared to DARC-positive individuals in both HIV negative (p = 0.0004) and HIV-1 infected (p<0.0001) study participants (S2 Table). A trend of higher monocyte counts were exhibited in HIV negative individuals compared to HIV infected subjects (p = 0.08) and positively correlated with ANCs irrespective of HIV status. Lymphocyte, eosinophil and basophil counts did not associate with either HIV status or DARC variant and did not associate with ANC (S2 Table). ANCs negatively correlated with plasma IL-7 (p = 0.004), IL-12 (p = 0.004) and IL-17 (0.009) in HIV negative persons (S3 Table), but these associations were absent during HIV infection. ANCs positively associated with IL-10 levels (p = 0.03) in HIV-infected individuals and MCP-1 in both HIV negative (p = 0.05) and HIV positive (p = 0.04) individuals (S3 Table).

**Table 1:** Clinical characteristics of study participants.

Characteristic	HIV Status			DARC Genotype					
				HIV- (n = 20)			HIV+ (n = 19)		
	HIV- (n = 20)	HIV+ (n = 19)	p value	DARC- (n = 12)	DARC+ (n = 8)	p value	DARC- (n = 10)	DARC+ (n = 9)	p value
Age, years	21	23	0.05	21	22	0.09	23	23	0.59
	(19–22)	(20–24)		(19–22)	(20–23)		(20–23)	(20–24)	
Neutrophil, x10^3^ cells/mm^3^	3.81	2.45	0.08	2.59	4.99	0.0004	1.75	4.34*	<0.0001
	(2.27–4.76)	(1.72–4.19)		(2.13–3.39)	(4.48–5.73)		(1.59–2.21)	(3.27–5.45)*	
CD3 count, cells/mm^3^	1545	1585	0.79	1527	1622	0.97	1578	1713	0.24
	(1368–1885)	(1140–2038)		(1426–1716)	(1243–1973)		(1079–1738)	(1432–2442)	
CD4 count, cells/mm^3^	883	653	0.0025	883	918	0.70	608	709	0.44
	(710–1051)	(487–779)		(720–981)	(693–1066)		(487–759)	(533–845)	
Viral Load, RNA copies/ml	na	9100	na	na	na	na	9300	7200	0.90
		(1400–21000)					(3275–23250)	(750–70500)	

Data is represented as median (IQR).

*ANC median and IQR were calculated from 8 participants (ANC was not available for 1 participant). All HIV+ participants were antiretroviral therapy naïve. Abbreviations: DARC, Duffy Antigen Receptor for Chemokines; ANC, Absolute Neutrophil Count; IQR, Interquartile range; n, number; na, not applicable. Female participants: n = 38, Male participants: n = 1.

### Marked reduction of total NK cell counts in DARC-null HIV negative individuals

**Fig 1:**
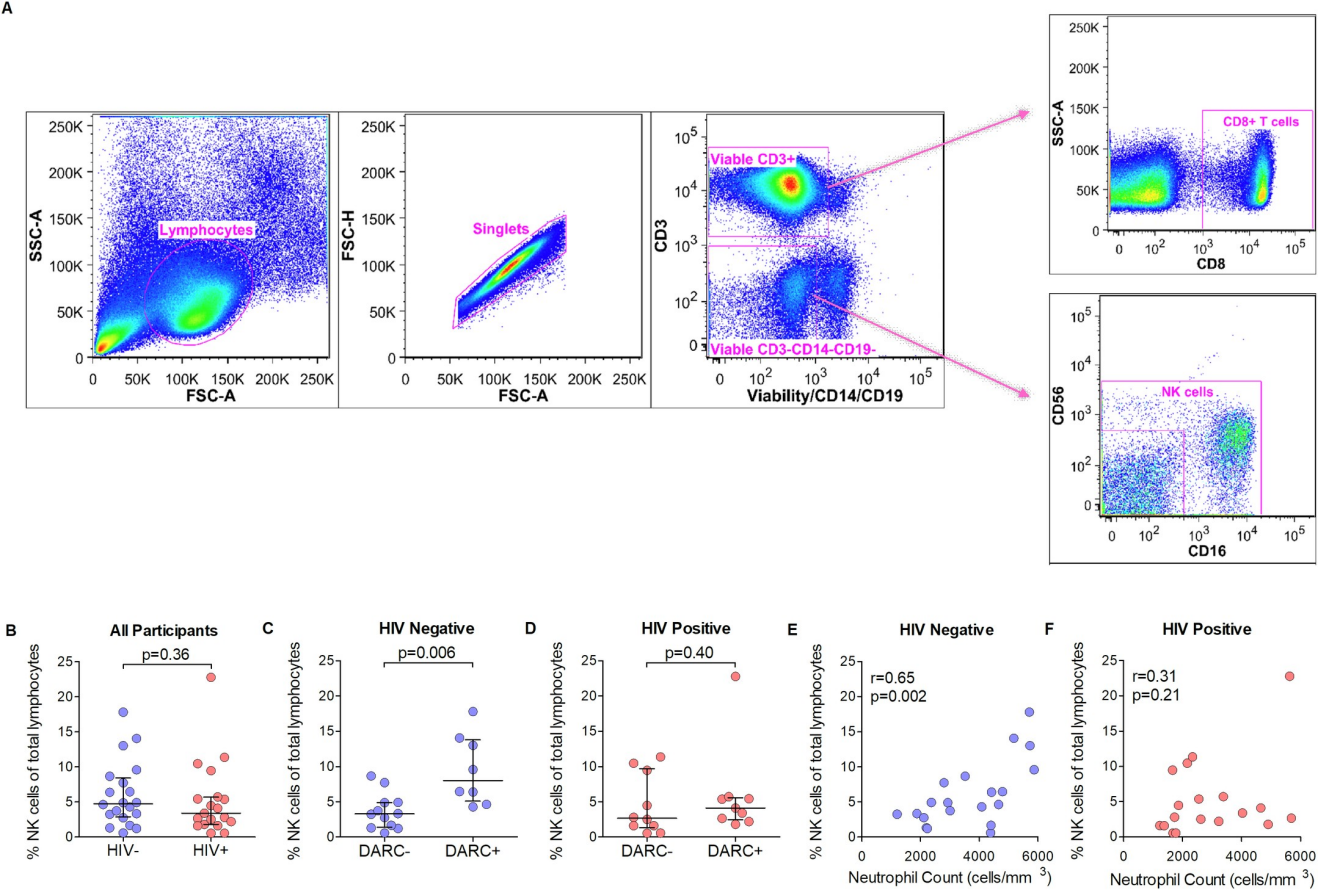
Total NK cell frequencies. (A) Representative gating strategy employed for the identification of total NK cells and CD8+ T cells from lymphocytes using multi-parametric flow cytometry. Side scatter area (SSC-A) vs forward scatter area (FSC-A) was used to identify lymphocytes followed by exclusion of doublets by forward scatter height (FSC-H) vs FSC-A, and exclusion of non-viable cells, B-cells and monocytes by use of viability dye and anti-CD19 and anti-CD14 monoclonal antibodies. Cytolytic T cells were identified using anti-CD3 and anti-CD8 monoclonal antibodies, whilst NK cells were identified as CD3 negative and CD56 positive and/or CD16 positive cells. (B) NK cell frequency as a percentage of total viable lymphocytes in all participants. NK cell frequency as a percentage of total viable lymphocytes stratified by DARC genotype in HIV negative (C) and HIV positive (D) individuals. Correlations between NK cells as a percentage of total lymphocytes and ANCs are shown in HIV negative (E) and HIV positive (F) individuals. Dots indicate individual data points. Medians are indicated and extended to interquartile range with whiskers. Abbreviations: DARC, Duffy antigen receptor for chemokines; ANC, Absolute neutrophil count; r, Spearman rho.

To gain insight of the circulating NK cell and CD8+ T cell populations in the context of DARC-null persons, NK cells were quantified based on the absence of CD3, CD14 and CD19 and the presence of CD56 and/or CD16 surface markers, while the frequencies of cytolytic T cells were measured based on the presence of CD3 and CD8 surface markers as proportions of the total viable lymphocyte population (Fig 1A). Total NK cell frequencies observed here were comparable between HIV negative and HIV-1 chronically infected persons (median frequency of 4.76% and 3.39% respectively, Fig 1B). In HIV negative individuals, subsequent analysis by DARC status indicated strikingly lower total NK cell frequencies in DARC-null individuals compared to DARC-positive individuals (p = 0.006, Fig 1C). Total NK cell frequencies also correlated with ANCs in HIV negative individuals (p = 0.002, Fig 1E). In contrast, assessment of HIV-infected individuals showed no differences in total NK cell frequencies when grouped by DARC status or ANCs (Fig 1D and 1F). Interestingly, IL-12 and G-CSF levels correlated significantly with lower NK cell frequencies in HIV negative persons (p = 0.02 and p = 0.009 respectively, S3 Table), but this association was lost in HIV infection. Measurement of CD8+ T cells indicated significantly higher frequencies during chronic HIV-1 infection (p = 0.001), but this difference was lost upon stratification by DARC and ANCs in HIV negative and HIV-1 positive persons respectively (S5 Table). Frequency data was verified using absolute NK cell and CD8+ T cell counts calculated from total white blood cell count and differentials at the time of sampling. Absolute counts showed similar significant differences in NK cell counts between DARC-null and DARC-positive HIV negative individuals, although to a lesser extent (S1 Fig). Taken together, these data indicate that the DARC-null trait and lower ANCs are associated with reduced peripheral NK cell counts in HIV negative persons only, whilst cytolytic CD8+ T cell frequencies were unaffected by the absence of DARC or lower ANCs irrespective of HIV status.

### Absence of DARC is associated with a less differentiated NK cell profile in HIV negative individuals

**Fig 2:**
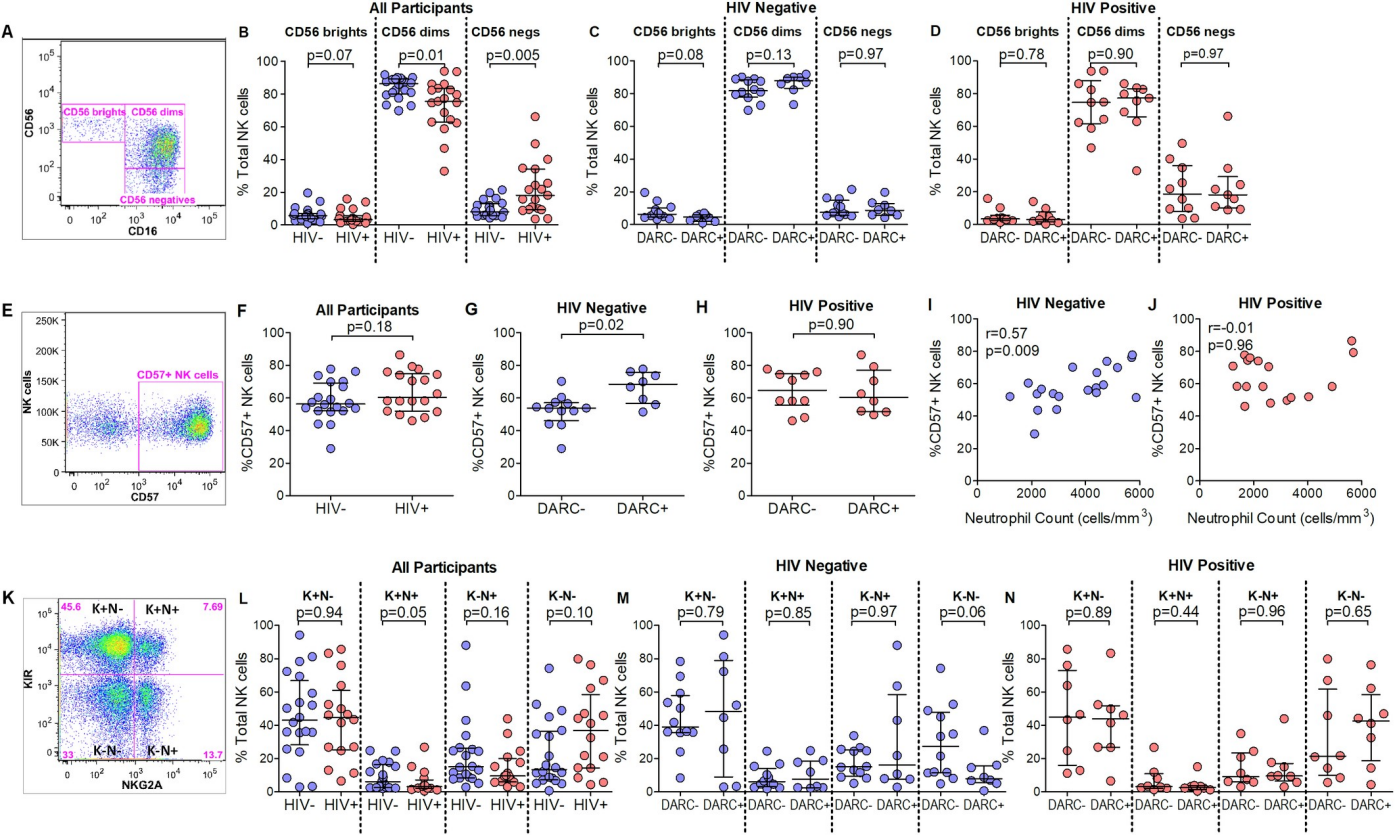
NK cell subset frequencies and markers of differentiation. (A) Total NK cells grouped by relative CD56 and CD16 expression into CD56 bright, CD56 dim and CD56 negative subsets. (B) NK cell subset frequencies in all participants. NK cell subset frequencies stratified by DARC genotype in HIV negative (C) and HIV positive (D) individuals. (E) Gating strategy for CD57 expressing NK cells as a percentage of total NK cells. (F) CD57 expressing NK cells as a percentage of total NK cells in all participants. CD57 expressing NK cells as a percentage of total NK cells stratified by DARC genotype in HIV negative (G) and HIV positive (H) individuals. Correlations between CD57 expressing NK cells and ANCs are shown in HIV negative (I) and HIV positive (J) individuals. (K) Representative strategy employed for the identification of KIR and/or NKG2A expressing NK cells. (L) KIR/NKG2A subsets as a percentage of total NK cells in all participants. KIR/NKG2A subsets further stratified by DARC genotype in HIV negative (M) and HIV infected (N) individuals. Dots indicate individual data points. Medians are indicated and extended to interquartile range with whiskers. Abbreviations: DARC, Duffy antigen receptor for chemokines; K+N-, KIR+NKG2A- NK cell subset; K+N+, KIR+NKG2A+ NK cell subset; K-N+, KIR-NKG2A+ NK cell subset; K-N-, KIR-NKG2A- NK cell subset.

NK cells are a phenotypically and functionally heterogeneous population, and proceed through several stages of development [27]. Various models were used to measure the maturation / differentiation status of NK cells. Firstly, NK cell subset composition was assessed based on the relative expression of CD56 and CD16, and identified as cytolytically mature CD56 dim (CD56+CD16+), cytokine producing CD56 bright (CD56+CD16-) or dysfunctional CD56 negative (CD56-CD16+) cells [28] (Fig 2A). In accordance with other studies our data indicated higher frequencies of the CD56 dim population in HIV negative individuals compared to HIV-infected donors (p = 0.01, Fig 2B), and elevated CD56 negative NK cell subset counts were observed during HIV-1 infection (p = 0.005, Fig 2B). Although NK cell subset frequencies were not significantly different when stratified by DARC genotype irrespective of HIV status (Fig 2C and 2D), a weak trend was noted towards higher frequencies of immature CD56 bright cells in DARC-null compared to DARC-positive HIV negative donors (p = 0.08, Fig 2C). In addition, a negative correlation was observed between CD56 bright frequencies and ANCs in HIV negative individuals (p = 0.05, S4 Table). Next we quantified NK cell CD57 surface expression (Fig 2E) as a marker of terminal differentiation and potent cytotoxicity [29]. HIV negative and HIV infected persons here demonstrated comparable CD57 expression on NK cells (Fig 2F). In HIV negative participants, CD57 expression was considerably lower in DARC-null compared to DARC-positive individuals (p = 0.02, Fig 2G), and CD57 expression strongly correlated with ANC (p = 0.009, Fig 2I). In contrast, no differences in CD57 expression were observed in HIV infected individuals based on DARC genotype (Fig 2H) or ANC (Fig 2J). Finally, NK cells were evaluated using the KIR and NKG2A model (Fig 2K) which delinates NK cell differentiation based on a stepwise decrease of NKG2A expression and acquisition of KIRs [30]. Lower frequencies of the cytolytic KIR+NKG2A+ subset were detected in HIV infected persons compared to uninfected individuals (p = 0.05, Fig 2L). HIV negative donors with the DARC-null trait displayed a trend of higher frequencies of the hypo-responsive KIR-NKG2A- subset (p = 0.06, Fig 2M) and ANCs were negatively associated with this KIR-NKG2A- subset (p = 0.03, S4 Table). No differences were indicated in any of the KIR/NKG2A subset frequencies in the HIV infected group when assessed by DARC (Fig 2N).

CD57 expression was also used to determine cellular senescence on CD8+ T cells [29, 31]. We observed no differences in CD57 expression on CD8+ T cells between HIV negative and positive individuals (p = 0.16). Furthermore, no differences in CD57 expression on CD8+ T cells were evident by DARC status in HIV negative or HIV positive individuals (S5 Table). Taken together, the data suggests similar frequencies of fully differentiated NK cells in HIV negative and HIV-1 infected persons. Interestingly, analysis by NK cell subset, CD57 expression and KIR/NKG2A analysis demonstrated a phenotype skewed toward an immature profile that was noted in HIV negative subjects in the absence of DARC and associated with ANCs; however this was lost in chronic HIV infection. In contrast, we noted no measurable differences of CD8+ T cell senescence by HIV or DARC status in these individuals.

### NK cell and CD8+ T cell activation and exhaustion markers differ by HIV status but not by DARC status

**Fig 3:**
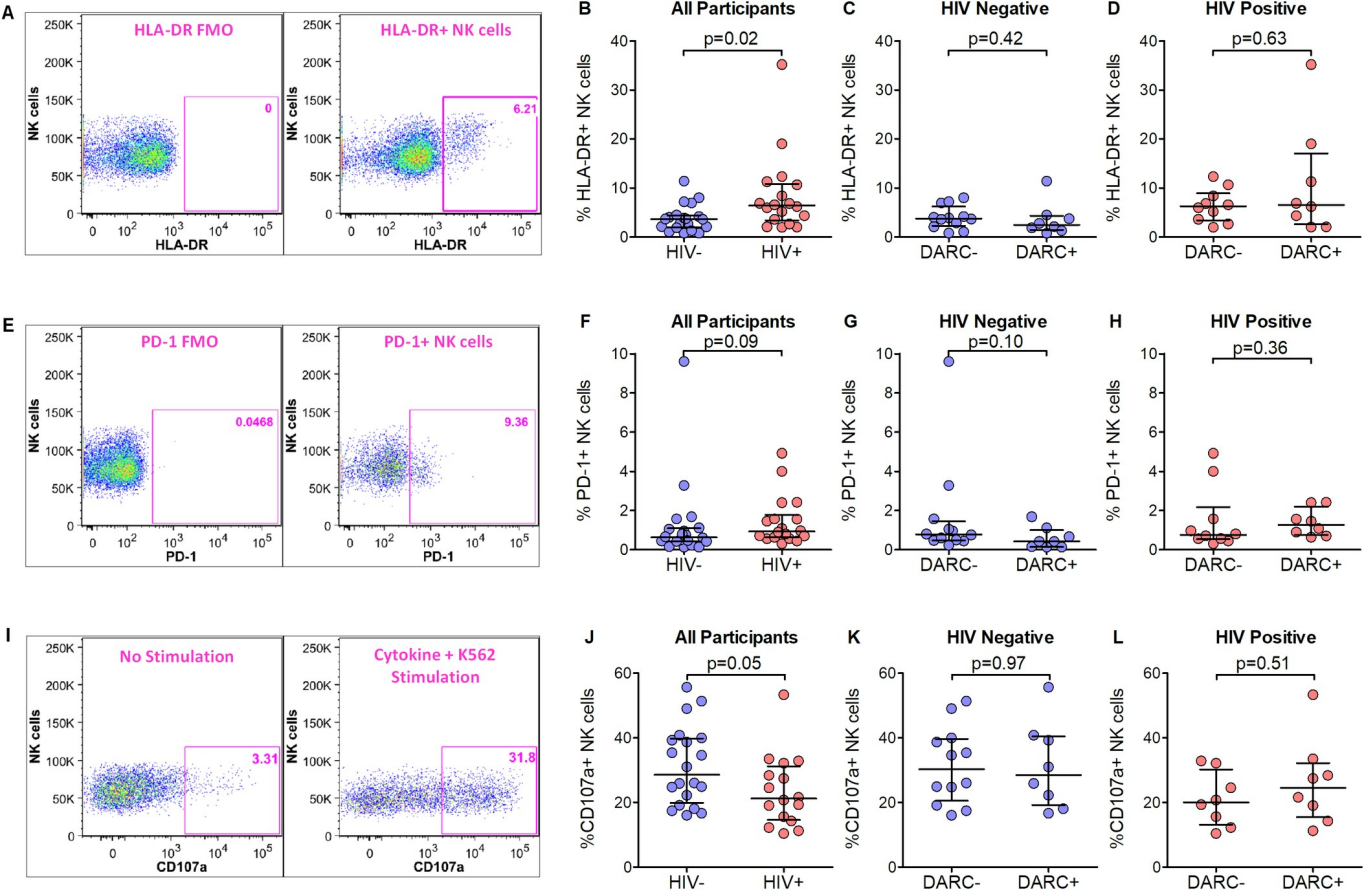
NK cell activation, exhaustion and degranulation. (A) Gating strategy of activation marker HLA-DR on total NK cells. (B) Expression of activation marker HLA-DR on total NK cells in all participants. Expression of activation marker HLA-DR grouped by DARC genotype on total NK cells in HIV negative (C) and HIV positive (D) individuals. (E) Gating strategy of exhaustion marker PD-1 on total NK cells. (F) Expression of exhaustion marker PD-1 on total NK cells in all participants. Expression of PD-1 marker grouped by DARC genotype on total NK cells in HIV negative (G) and HIV positive (H) individuals. (I) Gating strategy of degranulation marker CD107a on total NK cells. (J) Expression of degranulation marker CD107a on total NK cells in all participants. CD107a expression on total NK cells stratified by DARC genotype in HIV negative (K) and HIV positive (L) individuals. Dots indicate individual data points. Medians are indicated and extended to interquartile range with whiskers. Abbreviations: DARC, Duffy antigen receptor for chemokines.

Next we aimed to determine whether NK cell or CD8+ T cell activation and exhaustion *ex vivo* differed by HIV status and DARC genotype by assessing previously reported phenotypic markers HLA-DR (Fig 3A) and PD-1 [32] (Fig 3E) respectively. We hypothesised that the absence of DARC would contribute to a modified cellular phenotype. Consistent with a previous report [33], chronic NK cell activation as measured by HLA-DR was evident in HIV infected persons compared to HIV negative controls (p = 0.02, Fig 3B). In addition, NK cells from HIV-infected persons also showed a trend of higher expression of exhaustion marker PD-1 compared to uninfected individuals (p = 0.09, Fig 3F). However, NK cell expression of HLA-DR and PD-1 was not affected by DARC genotype (Fig 3C, 3D, 3G and 3H) and did not significantly associate with ANCs (S4 Table) in HIV negative persons or HIV infected individuals. We measured CD8+ T cell activation by assessing co-expression of CD38 and HLA-DR and exhaustion by assessing PD-1 expression [34]. As expected, the frequency of CD38/HLA-DR expressing CD8+ T cells were significantly higher among HIV-1 infected subjects compared to HIV-1 negative subjects (p<0.0001). Furthermore, higher PD-1 expression on CD8+ T cells was displayed in HIV-1 positive individuals compared to HIV uninfected persons (p = 0.05). However, neither CD38/HLA-DR or PD-1 expression analysis displayed differences by DARC status and did not associate with ANCs regardless of HIV status (S5 Table). Taken together these data supports previous findings on NK cell and CD8+ T cell activation and exhaustion profiles during HIV infection. Contrary to our hypothesis the data revealed that neither DARC genotype nor ANC had an impact on activation and exhaustion status of NK cells or cytolytic CD8+ T cells.

### No differences in effector activity of NK cells or CD8+ T cells in individuals with the DARC-null polymorphism

To investigate the impact of DARC genotype and ANC on NK and CD8+ T cell functionality we assessed degranulation (CD107a expression; Fig 3I for NK cell gating strategy) and TNF-α and IFN-ɣ cytokine production in PBMCs following overnight culture with appropriate stimulation. Our results indicated higher CD107a expression on total NK cells from HIV negative compared to HIV infected individuals upon stimulation with the K-562 cell line (p = 0.05, Fig 3J), while no differences in the proportion of TNF-α and IFN-ɣ secreting NK cells were evident in HIV infection (S4 Table). Further assessment detected no differences in degranulation (Fig 3K and 3L) or cytokine production (S4 Table) in NK cells when stratified by DARC trait.

Cytolytic CD8+ T cells from HIV-1 infected individuals exhibited an increase in CD107a expression upon stimulation with Gag (p = 0.03) and gp41 (p = 0.01) peptide pools respectively compared to HIV negative subjects. In contrast, no differences were noted in CD107a expression on CD8+ T cells during HIV infection upon stimulation with gp120 peptide (p = 0.17). Assessment of cytokine production indicated higher IFN-ɣ production in CD8+ T cells following stimulation with Gag (p = 0.02) and gp41 (p = 0.004) in HIV infected individuals; whilst no differences in TNF-α production were evident by HIV status. Stratification by DARC genotype indicated that CD8+ T cell degranulation and cytokine production were unaffected irrespective of HIV status or HIV peptide stimulation (S5 Table). Taken together our data indicated altered degranulation activity of NK cells and CD8+ T cells in HIV infection. However, the results generated here suggest that cytotoxic cellular activities are not altered by the DARC-null polymorphism or reduced ANCs irrespective of HIV status.

### Reduced survival of activated NK cells in HIV infected individuals with DARC-null genotype, whilst both NK cell and CD8+ T cell proliferation are unaffected by the absence of DARC

**Fig 4:**
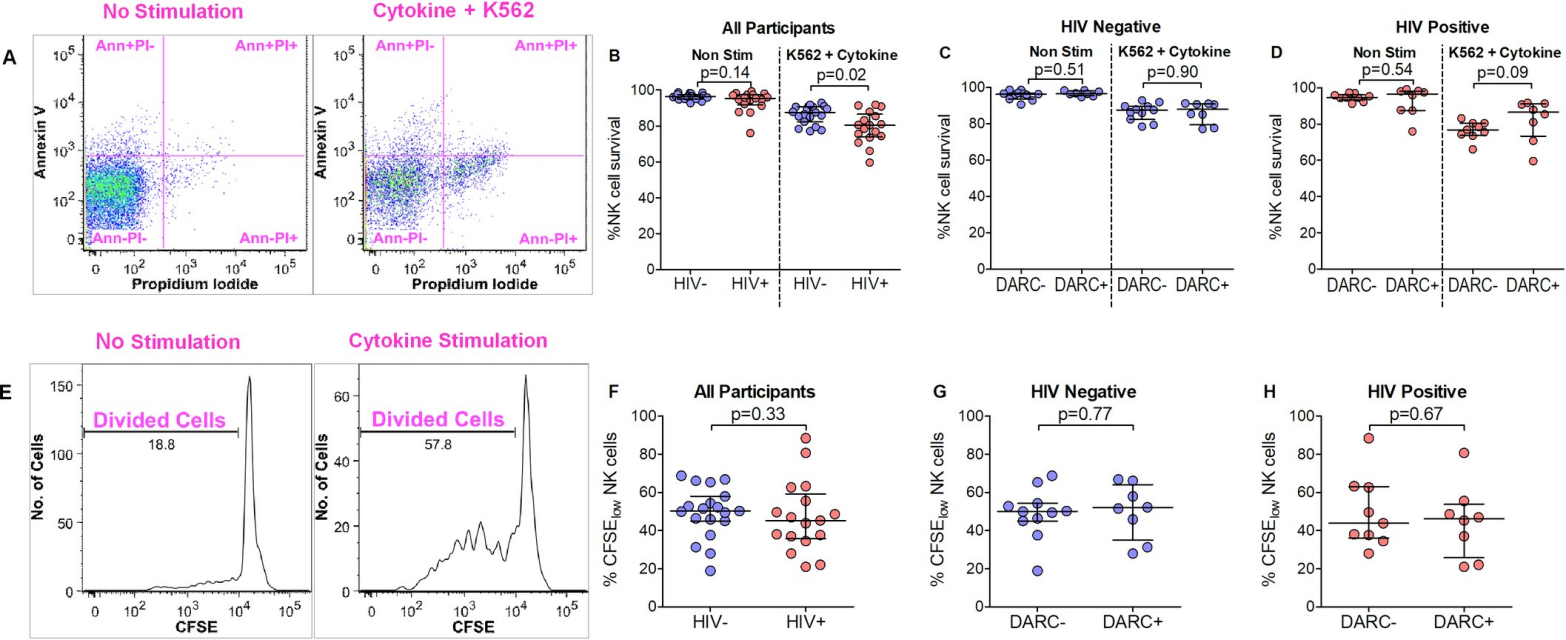
NK cell survival and proliferation measurement. (A) Gating strategy of Annexin V and propidium iodide on total NK cells. NK cells that survived were identified as Annexin (Ann)-PI-. (B) Percentage of NK cell survival for unstimulated and K-562+cytokine stimulated cells in all participants. Participants were further stratified to show percentage of NK survival for unstimulated and K-562+cytokine stimulated cells in HIV negative (C) and HIV positive (D) individuals by DARC trait. (E) Gating strategy of CFSE labeling on total NK cells. (F) Percentage of NK cells divisions (CFSE low) in all participants. Participants were further stratified to show percentage of NK cells divided in HIV negative (G) and HIV positive (H) individuals by DARC trait. Dots indicate individual data points. Medians are indicated and extended to interquartile range with whiskers. Abbreviations: DARC, Duffy antigen receptor for chemokines; CFSE, carboxyfluorescein succinimidyl ester.

Our earlier data showed that the DARC-null polymorphism and lower ANCs were associated with lower peripheral NK cell numbers in HIV negative individuals which may arise from modulation of NK cell homeostatic activities. We therefore investigated the possible influence of the DARC-null trait on NK cell survival using annexin and propidium staining (Fig 4A) and proliferation using CFSE labeling (Fig 4E); and we further examined possible alterations to CD8+ T cell proliferation in the context of the DARC genotype. We observed similar median frequencies of NK cell survival of 96% in HIV negative and 95% in HIV-infected individuals when cells were left untreated, although examination by NK cell subset showed that CD56 bright cells were highly reduced in HIV infection (p = 0.009, S2A Fig). Median frequencies of total NK cell survival were significantly higher in HIV negative (87%) compared to HIV infected individuals (80%) following cellular activation with K-562 cell line and cytokines (p = 0.02, Fig 4B) and this was attributed to a loss of CD56 bright and CD56 dim cells in HIV-infected individuals following stimulation (S2A Fig). HIV negative individuals grouped by DARC trait showed no differences in the frequency of NK cell survival following untreated or stimulated conditions (Fig 4C, S2B Fig). A trend of lower NK cell survival was noted in activated NK cells from DARC-null individuals compared to DARC-positive participants (p = 0.09, Fig 4D) during HIV infection owing to lower survial of CD56 dim and CD56 negative cells (S2C Fig). Further examination showed no association between NK cell survival and ANCs in either HIV negative or HIV infected individuals (S4 Table).

Cell proliferation due to activation was measured by subtracting CFSE divisions in unstimulated cells from CFSE divisions in either cytokine stimulated NK cells or gag peptide stimulated CD8+ T cells. We hypothesised that HIV infected individuals would have higher levels of proliferating NK cells and CD8+ T cells; and that presence of the DARC-null polymorphism would adversely impact proliferation of both NK and CD8+ T cells. In contrast to our hypothesis, we observed similar levels of NK cell expansion following cytokine induction in HIV negative and HIV infected subjects where median NK cell proliferation frequencies were 50% and 45% respectively (Fig 4F). Moreover, we detected no differences in NK cell proliferation by DARC trait in both HIV negative (Fig 4G) and chronically infected subjects (Fig 4H). In contrast, we did observe higher proliferation of CD8+ T cells in HIV-1 infected individuals (p = 0.05, S5 Table); however, no differences were detected in the proliferative capacity of CD8+ T cells by DARC status in either HIV negative or HIV positive individuals (S5 Table).

Together the data suggests that NK cell survival is unaffected by DARC status in HIV negative individuals. However, survival of activated NK cells is reduced during HIV infection, and this is more evident in DARC-null individuals. Our results showed no differences in NK cell proliferation by HIV status; while increased cellular proliferation in CD8+ T cells was exhibited following activation. Furthermore the data indicates that, contrary to our hypothesis, DARC status and ANC levels do not impact NK cell or CD8+ T cell division irrespective of HIV infection.

## Discussion

DARC-null linked neutropenia is associated with increased HIV-1 infection risk, however its impact on HIV pathogenesis is contested. Notably, cytolytic lymphocyte priming is dependent on neutrophil interaction in various anatomical compartments. Here we assessed the impact of the DARC-null trait and lower ANCs on peripheral NK and CD8+ T cell profiles in black individuals from the HIV prevalent KwaZulu-Natal region in South Africa. We report that the DARC-null trait and lower ANCs are associated with reduced NK cell counts in HIV negative individuals. These NK cells displayed an immature phenotype which surprisingly did not translate to evident cellular dysfunction. Interestingly, in comparison to uninfected persons, no differences were noted in NK cell profiles upon DARC genotype stratification in HIV infected study participants. Furthermore, no apparent phenotypic or functional changes were observed in cytolytic T cells by DARC genotype irrespective of HIV status.

During chronic HIV infection NK cell counts are typically reduced and subset redistribution is associated with viremia [35–37]. Highly differentiated NK cells [38, 39] with elevated activation and exhaustion levels [37, 40, 41] and increased cytotoxicity and antiviral cytokine release [42, 43] are reported to be associated with disease progression to AIDS. Similarly, we noted altered NK subset distribution and higher expression levels of activation and exhaustion markers in HIV infected individuals. In contrast to past studies, NK cells exhibited no differences in CD57 expression, lower degranulation capacity and unaltered cytokine release during chronic HIV infection. Inclusion of the CD56 negative subset in our analyses could explain the disparate observations. Previously, we described reduced CD57 expression on CD56 negative NK cells during chronic infection [44]. Lower viral loads and accumulation of the dysfunctional CD56 negative subset could also drive inferior cytolytic activity in HIV infected individuals [35, 44]. Reduced survival of activated NK cells was observed in HIV infected individuals, which has previously been attributed to HIV envelope protein exposure [45–47]. No observable differences were exhibited in cytokine-induced NK cell proliferation. Since viremia contributes to either NK cell expansion via heightened IL-15 serum levels [48, 49] or proliferative suppression due to HIV encoded proteins [46, 50, 51], low viral loads measured in participants here could explain the absence of differences in proliferation levels by HIV status.

In line with past studies, HIV-1 infected individuals exhibited significantly higher cytolytic T cell counts [52], elevated activation marker expression [53–55], cellular exhaustion due to persistent activation; although to a lesser degree than previously reported [34, 56] and higher levels of CD8+ T cell degranulation and IFN-γ production in response to HIV-1 peptides [57–59]. Unexpectedly, accelerated cytolytic T cell senescence as measured by CD57 expression [60] was not found in HIV-1 infected persons. Interestingly, Bowers and colleagues ascertained that HIV-1 virion induced expression of PD-L1 on neutrophils positively associated with PD-1 and CD57 expression on CD8+ T cells [61]. Lower PD-1 levels and unaltered CD57 expression on CD8+ T cells could be due to the low viral loads exhibited by study participants in our study. Further to this we observed a trend of higher proliferation of CD8+ T cells in HIV-1 infected individuals as supported by previous reports of activated T cell accumulation in an attempt to clear virus [62].

The DARC-null polymorphism is the genetic determinant of ethnic neutropenia in African ancestry populations [5]. Consistent with our previous report [16], the DARC-null genotype significantly associated with reduced ANCs irrespective of HIV status. We recently reported unimpaired neutrophil functionality in individuals with the DARC-null trait [16]. Here we explored the impact of the DARC-null polymorphism on cytolytic cell priming. Jaeger and collegues described comparable NK cell counts in neutropenic and healthy persons; interestingly increased cell death sensitivity was counteracted by hyper-proliferative capacity in subsequent neutropenic mouse models [21]. Dissimilarly, we observed reduced NK cell counts in DARC-null HIV negative individuals with lower ANCs, suggesting unbalanced homeostatic activities leading to lower circulating NK cells. However, no differences were measured in NK cell survival or proliferation in DARC-null uninfected persons; relatively higher neutrophil counts in DARC-null individuals compared to other neutrophil disorders could explain these findings since observable NK cell modulation is usually detected during severe neutrophil deficiency [21]. Factors including diminished NK cell release from the bone marrow or altered trafficking of NK cells between tissues and circulation could explain the reduced NK cell frequencies [63]. Interestingly, NK cell frequencies negatively associated with circulatory G-CSF in HIV negative persons. A few studies have described a decrease in the percentage of NK cells of total lymphocytes in the bone marrow and peripheral blood following G-CSF administration amongst healthy haematopoietic stem cell transplantation donors [64, 65]. It is possible that a direct link seen on NK cells in previous studies might also be noticed here. However, reasons why this defect would be notable in HIV uninfected and not in HIV infected individuals requires further exploration and is most likely associated with overall defects associated with HIV infection.

Neutropenic individuals previously exhibited higher frequencies of immature CD56 bright cells coupled with impaired IFN-γ release [21]. Although HIV uninfected DARC-null individuals exhibited a less mature NK cell repertoire suggestive of hypo-responsiveness, further investigation detected no differences in activation or exhaustion profiles and cytotoxic mechanisms by DARC status. Re-education of hypo-responsive NK cells is probable following specific cytokine stimulation [30]. Thus *in vitro* stimulation possibly promoted further differentiation and typical cytolytic activity of NK cells from DARC-null individuals. Interestingly, NK cell variations that were detected by DARC trait in HIV negative donors were not observed in HIV-1 infected individuals, implying that DARC-null trait may not influence NK cell responses in chronic infection. It is plausible that any advantage that DARC-positive individuals are predisposed to is masked by the general leukopenia commonly detected in chronic disease [66]. Alternatively, these advantages could be lost in untreated chronic HIV-1 infection.

The effects of neutrophil deficiency on T cell dynamics in the context of HIV infection is unclear. Functional suppression of T cells by neutrophils via cell-cell communication and neutrophil reactive oxygen species has been characterised in chronic infection [61]. Contrary to our hypothesis of T cell profile modulation in the presence of the DARC-null polymorphism, evidence of phenotypic or functional alteration were unapparent. Neutrophils have been reported to provide regulatory cues for efficient virus-specific CD8+ T cell migration and effector functionality during tracheal influenza infection in mice [67]. Incidentally, cutaneous herpes simplex virus type 1 infection in mice showed unaffected priming and expansion of CD8+ T cells in the lymph node following neutrophil depletion; thus suggesting that neutrophil immune-modulation of T cells may be context and pathogen specific [68].

This pilot study was limited by a small sample size which may mask subtle differences. The cohort was predominantly female and data for confounding factors such as menstrual cycle and hormonal levels were not available. These factors can contribute to variation of immune cell function [69, 70] and could influence our results. DARC-null individuals had low ANCs but were not neutropenic as defined by the ANC threshold of <1500 cells/mm^3^. ANCs were taken cross-sectionally at the time of sampling. Without longitudinal readings we cannot exclude the possibility that ANCs may have been affected by minor inflammatory reactions that can temporarily increase ANCs in neutropenic individuals. Although testing negative for ART, some HIV infected patients had viral loads <1000 RNA copies/ml; and sample collection from ART naïve subjects became challenging due to the implementation of the HIV universal test and treat policy. Furthermore, time of infection was indeterminable, and without pre- and post-infection samples, we were unable to fully assess the impact of DARC-null genotype on cytolytic cells in chronic HIV-1 infection. We also recognize that our flow cytometry assay panels are not exhaustive and an in-depth assessment of other phenotypic and functional markers would be beneficial in future studies. Overall our findings of reduced NK cells may impart dampened responses in DARC-null HIV negative individuals, and may result in greater risk to viral infections in these individuals [71, 72]. Nevertheless, it is encouraging that from the data presented here, functional impairment of NK or cytolytic T cells was not evident in DARC-null individuals irrespective of HIV status. This may suggest that effector cell priming is either not heavily impacted or is sufficiently compensated through alternative mechanisms [73]. In conclusion, our findings do not support the proposed model of differential cytolytic cell priming and function in HIV infected DARC-null populations [6], suggesting that the effects of DARC-null neutropenia are not as pronounced as reported for other neutrophil deficiencies [7] and may thus not contribute to HIV disease progression in African populations.

## Supporting information

S1 Fig NK cell and CD8+ T cell absolute counts. (A) Absolute NK cell counts in all participants. Absolute NK cell counts stratified by DARC genotype in HIV negative (B) and HIV positive (C) individuals. Correlations between absolute NK cell counts and ANCs are shown in HIV negative (D) and HIV positive (E) individuals. (F) Absolute CD8+ T cell counts in all participants. Absolute CD8+ T cell counts stratified by DARC genotype in HIV negative (G) and HIV positive (H) individuals. Correlations between absolute CD8+ T cell counts and ANCs are shown in HIV negative (I) and HIV positive (J) individuals. Dots indicate individual data points. Medians are indicated and extended to interquartile range with whiskers. Abbreviations: DARC, Duffy antigen receptor for chemokines; ANC, Absolute neutrophil count; r, Spearman rho.
(PDF)

S2 Fig NK cell subset survival. (A) Percentage of survival by NK cell subset for unstimulated and K-562+cytokine stimulated cells in all participants. Participants were further stratified to show percentage of survival by NK cell subset for unstimulated and K-562+cytokine stimulated cells in HIV negative (B) and HIV positive (C) individuals by DARC trait. Dots indicate individual data points. Medians are indicated and extended to interquartile range with whiskers. Abbreviations: DARC, Duffy antigen receptor for chemokines.
(PDF)

S1 Table List of antibodies used for multi-parametric flow cytometry. (PDF)

S2 Table Peripheral blood cell counts of study participants. Data is represented as median (IQR). All PBC counts = x10^3^ cells/mm^3^. PBC count median and IQR were calculated from 18 HIV+ participants (PBC counts were not available for 1 participant). Abbreviations: DARC, Duffy Antigen Receptor for Chemokines; PBC, Peripheral blood cell; IQR, Interquartile range; n, number.
(PDF)

S3 Table Cytokine / chemokine association with ANC and absolute NK cell count. Data is represented as median (IQR). *Associations were calculated from 18 HIV+ participants (Full blood count was not available for 1 participant). Abbreviations: IQR, Interquartile range; n, number.
(PDF)

S4 Table NK cell profiles of study participants. Data is represented as median (IQR). Total NK cell data is presented as a percentage of the lymphocyte gate. Phenotype, functional, survival and proliferation data is presented as a percentage of total NK cells. *ANC associations were calculated from 18 HIV+ participants (ANC was not available for 1 participant). Low NK cell frequencies in some individuals made downstream gating of certain data unreliable. This data has been omitted from the analysis and are indicated as follows: ^#^Medians (IQR) were calculated from 18 HIV+ participants (8 HIV+DARC+); ^##^Medians (IQR) were calculated from 16 HIV+ participants (8 HIV+DARC- and 8 HIV+DARC+); ^###^Medians (IQR) were calculated from 19 HIV- (11 HIV-DARC- and 8 HIV-DARC+) and 17 HIV+ (9 HIV+DARC- and 8 HIV+DARC+) participants respectively. Abbreviations: DARC, Duffy Antigen Receptor for Chemokines; IQR, Interquartile range; n, number.
(PDF)

S5 Table CD8+ T cell profiles of study participants. Data is represented as median (IQR). CD8+ T cell data is presented as a percentage of the lymphocyte gate. Phenotype, functional and proliferation data is presented as a percentage of CD8+ T cells. *ANC associations were calculated from 18 HIV+ participants (ANC was not available for 1 participant). Abbreviations: DARC, Duffy Antigen Receptor for Chemokines; IQR, Interquartile range; n, number.
(PDF)
