# Supplementary figures and images for: The DARC-null trait is associated with moderate modulation of NK cell profiles and unaltered cytolytic T cell profiles in black South Africans
Source: PLoS One. 2020 Nov 19;15(11):e0242448. doi: 10.1371/journal.pone.0242448 (PMC7676658; doi:10.1371/journal.pone.0242448)

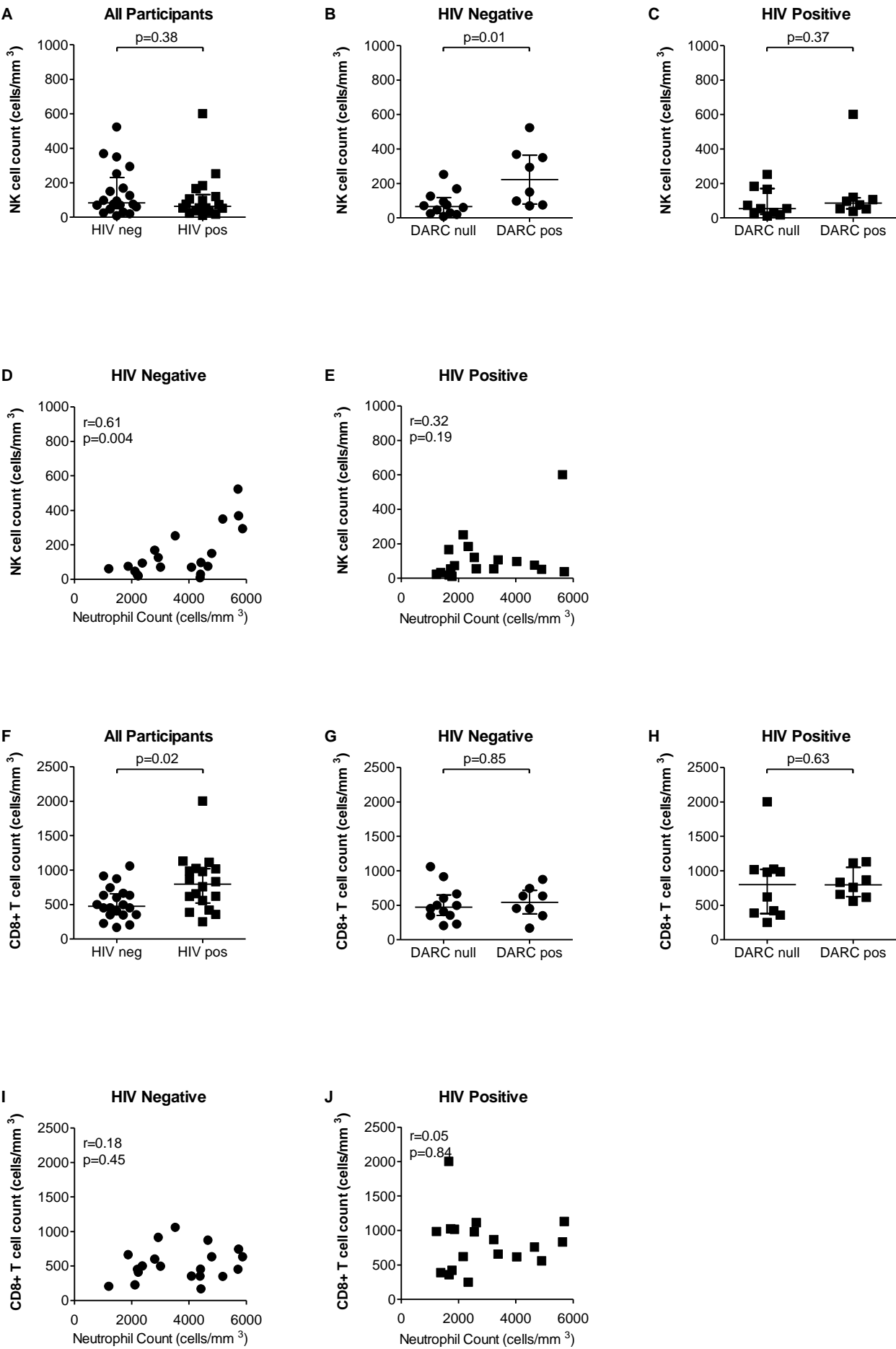

Supplement: S1 Fig — (A) Absolute NK cell counts in all participants. Absolute NK cell counts stratified by DARC genotype in HIV negative (B) and HIV positive (C) individuals. Correlations between absolute NK cell counts and ANCs are shown in HIV negative (D) and HIV positive (E) individuals. (F) Absolute CD8+ T cell counts in all participants. Absolute CD8+ T cell counts stratified by DARC genotype in HIV negative (G) and HIV positive (H) individuals. Correlations between absolute CD8+ T cell counts and ANCs are shown in HIV negative (I) and HIV positive (J) individuals. Dots indicate individual data points. Medians are indicated and extended to interquartile range with whiskers. Abbreviations: DARC, Duffy antigen receptor for chemokines; ANC, Absolute neutrophil count; r, Spearman rho. (PDF) [file pone.0242448.s001.pdf]

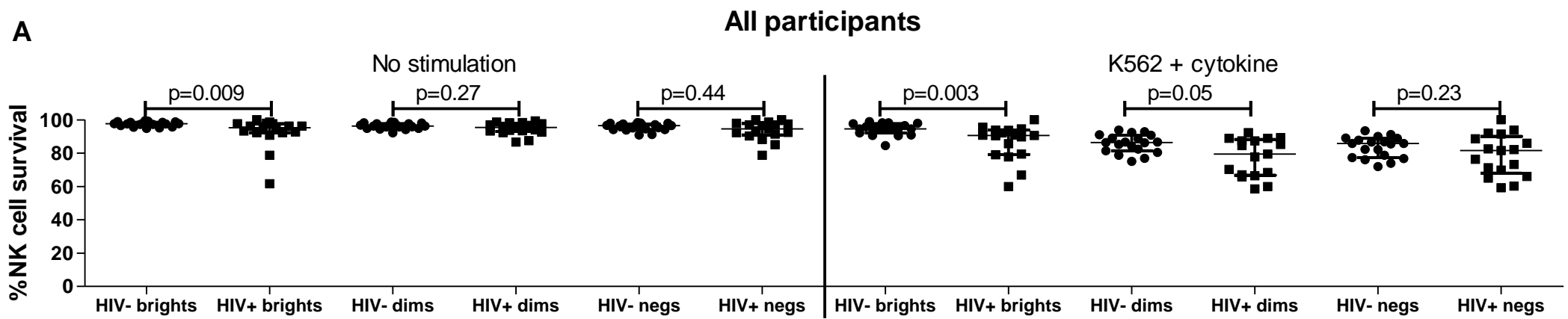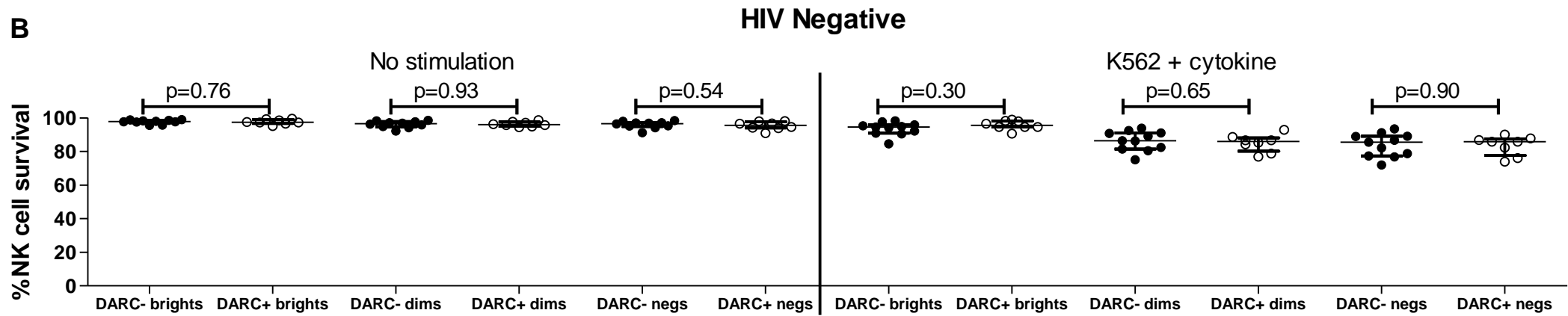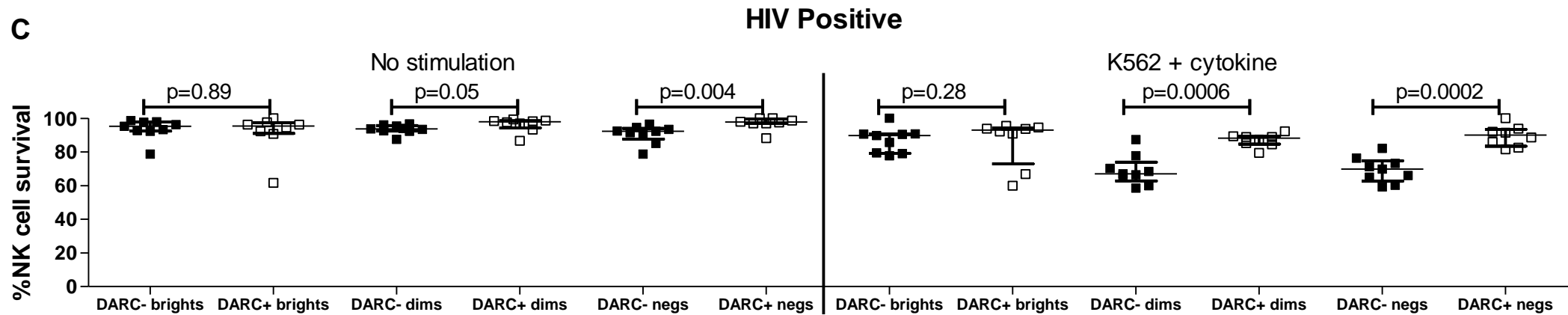

Supplement: S2 Fig — (A) Percentage of survival by NK cell subset for unstimulated and K-562+cytokine stimulated cells in all participants. Participants were further stratified to show percentage of survival by NK cell subset for unstimulated and K-562+cytokine stimulated cells in HIV negative (B) and HIV positive (C) individuals by DARC trait. Dots indicate individual data points. Medians are indicated and extended to interquartile range with whiskers. Abbreviations: DARC, Duffy antigen receptor for chemokines. (PDF) [file pone.0242448.s002.pdf]
