# Supplementary material for: The DARC-null trait is associated with moderate modulation of NK cell profiles and unaltered cytolytic T cell profiles in black South Africans
Source: PLoS One. 2020 Nov 19;15(11):e0242448. doi: 10.1371/journal.pone.0242448 (PMC7676658; doi:10.1371/journal.pone.0242448)
Supplement: S1 Table — (PDF) [file pone.0242448.s003.pdf]

Antigen	Fluorochrome	Antigen Type	Host Species	Clone	Manufacturer	Vol/sample (µl)
CD3	PE-CF594	Monoclonal	Mouse	UCHT1	BD Biosciences	2
CD3	APC	Monoclonal	Mouse	UCHT1	BD Biosciences	2
CD4	APC-Cy7	Monoclonal	Mouse	RPA-T4	Biolegend	1.5
CD8	PE-Cy7	Monoclonal	Mouse	SK1	BD Biosciences	4
CD8	Alexa-Fluor-700	Monoclonal	Mouse	RPA-T8	BD Biosciences	1.5
CD14	V500	Monoclonal	Mouse	M5E2	BD Biosciences	2
CD14	BV650	Monoclonal	Mouse	M5E2	Biolegend	2
CD19	V500	Monoclonal	Mouse	HIB19	BD Biosciences	2
CD19	BV650	Monoclonal	Mouse	HIB19	Biolegend	2
CD16	APC-Cy7	Monoclonal	Mouse	3G8	BD Biosciences	1.5
CD56	Alexa-Fluor-700	Monoclonal	Mouse	B159	BD Biosciences	1
CD69	FITC	Monoclonal	Mouse	FN50	BD Biosciences	5
CD38	PE	Monoclonal	Mouse	HB7	BD Biosciences	4
HLA-DR	BV711	Monoclonal	Mouse	L243	Biolegend	2
PD-1	BV421	Monoclonal	Mouse	EH12.2H7	Biolegend	2
CD57	APC	Monoclonal	Mouse	NK-1	BD Biosciences	2.5
CD158a	PE	Monoclonal	Mouse	HP-3E4	BD Biosciences	5
CD158b	PE	Monoclonal	Mouse	CH-L	BD Biosciences	5
CD158e1/e2	PE	Monoclonal	Mouse	Z27.3.7	Beckman Coulter	5
NKG2A	APC	Monoclonal	Mouse	Z199.1	Beckman Coulter	5
CD107a	PE-Cy5	Monoclonal	Mouse	H4A3	BD Biosciences	3.5
TNF-α	PerCP-Cy5.5	Monoclonal	Mouse	Mab11	Biolegend	5
IFN-γ	PE-Cy7	Monoclonal	Mouse	B27	Biolegend	5
