# Supplementary material for: The DARC-null trait is associated with moderate modulation of NK cell profiles and unaltered cytolytic T cell profiles in black South Africans
Source: PLoS One. 2020 Nov 19;15(11):e0242448. doi: 10.1371/journal.pone.0242448 (PMC7676658; doi:10.1371/journal.pone.0242448)
Supplement: S2 Table — Data is represented as median (IQR). All PBC counts = x103 cells/mm3. PBC count median and IQR were calculated from 18 HIV+ participants (PBC counts were not available for 1 participant). Abbreviations: DARC, Duffy Antigen Receptor for Chemokines; PBC, Peripheral blood cell; IQR, Interquartile range; n, number. (PDF) [file pone.0242448.s004.pdf]

Peripheral Blood Cell	HIV Status			DARC Genotype						ANC Association			
				HIV- (n=20)			HIV+ (n=18)			HIV- (n=20)		HIV+ (n=18)	
	HIV- (n=20)	HIV+ (n=18)	p value	DARC - (n=12)	DARC+ (n=8)	p value	DARC- (n=10)	DARC + (n=8)	p value	Spearman r	p value	Spearman r	p value
White cell count	6.69 (5.15-8.14)	5.41 (4.75-7.57)	0.11	5.96 (4.64-6.93)	8.01 (6.81-10.19)	0.0061	5.04 (3.87-5.41)	7.58 (6.00-8.20)	0.0004	0.86	<0.0001	0.91	<0.0001
Neutrophil count	3.81 (2.27-4.76)	2.45 (1.72-4.19)	0.08	2.59 (2.13-3.39)	4.99 (4.48-5.73)	0.0004	1.75 (1.59-2.21)	4.34 (3.27-5.45)	<0.0001	na	na	na	na
Lymphocyte count	1.93 (1.69-2.57)	2.00 (1.72-2.51)	0.97	1.91 (1.71-2.24)	2.41 (1.62-2.91)	0.62	1.94 (1.64-2.24)	2.36 (1.85-2.81)	0.27	0.32	0.17	0.23	0.35
Monocyte count	0.50 (0.37-0.70)	0.42 (0.35-0.48)	0.08	0.46 (0.36-0.53)	0.53 (0.42-0.87)	0.18	0.38 (0.31-0.45)	0.47 (0.40-0.56)	0.06	0.52	0.02	0.43	0.07
Eosinophil count	0.17 (0.08-0.50)	0.14 (0.06-0.31)	0.51	0.15 (0.06-0.31)	0.24 (0.10-0.61)	0.40	0.14 (0.09-1.09)	0.15 (0.05-0.26)	0.72	0.15	0.52	-0.01	0.96
Basophil count	0.02 (0.01-0.04)	0.03 (0.02-0.04)	0.39	0.02 (0.01-0.04)	0.03 (0.01-0.04)	0.55	0.03 (0.02-0.03)	0.04 (0.02-0.04)	0.26	-0.13	0.58	0.27	0.27
