# Supplementary material for: The DARC-null trait is associated with moderate modulation of NK cell profiles and unaltered cytolytic T cell profiles in black South Africans
Source: PLoS One. 2020 Nov 19;15(11):e0242448. doi: 10.1371/journal.pone.0242448 (PMC7676658; doi:10.1371/journal.pone.0242448)
Supplement: S3 Table — Data is represented as median (IQR). *Associations were calculated from 18 HIV+ participants (Full blood count was not available for 1 participant). Abbreviations: IQR, Interquartile range; n, number. (PDF) [file pone.0242448.s005.pdf]

Cytokine / Chemokine	ANC Association				NK cell Association			
	HIV- (n=20)		HIV+ (n=18)*		HIV- (n=20)		HIV+ (n=18)*	
	Spearman r	p value	Spearman r	p value	Spearman r	p value	Spearman r	p value
IL-1β	-0.37	0.12	0.09	0.73	-0.35	0.13	0.11	0.66
IL-2	0.01	0.97	0.35	0.15	-0.27	0.25	0.23	0.37
IL-4	-0.36	0.12	0.46	0.06	-0.34	0.15	0.20	0.43
IL-5	-0.07	0.78	0.07	0.78	0.002	0.99	-0.02	0.93
IL-6	0.24	0.32	0.32	0.20	0.16	0.50	-0.04	0.86
IL-7	-0.61	0.004	0.44	0.07	-0.27	0.25	0.32	0.20
IL-8	0.25	0.29	-0.25	0.31	0.15	0.54	-0.42	0.08
IL-10	-0.29	0.22	0.50	0.03	-0.25	0.29	0.25	0.31
IL-12	-0.61	0.004	0.30	0.23	-0.53	0.02	-0.19	0.45
IL-13	-0.44	0.06	0.36	0.14	-0.03	0.90	0.05	0.85
IL-17	-0.57	0.009	0.28	0.27	-0.12	0.62	-0.11	0.66
G-CSF	-0.29	0.21	0.23	0.36	-0.57	0.009	0.09	0.71
GM-CSF	-0.30	0.20	0.07	0.78	-0.22	0.35	-0.02	0.93
MCP-1	0.45	0.05	0.49	0.04	0.20	0.39	-0.14	0.57
MIP-1β	0.42	0.07	0.02	0.94	0.16	0.50	0.09	0.72
TNF-α	0.02	0.92	0.12	0.64	-0.06	0.80	0.16	0.52
IFN-γ	-0.10	0.68	0.29	0.25	0.04	0.85	-0.03	0.92
