# Supplementary material for: The DARC-null trait is associated with moderate modulation of NK cell profiles and unaltered cytolytic T cell profiles in black South Africans
Source: PLoS One. 2020 Nov 19;15(11):e0242448. doi: 10.1371/journal.pone.0242448 (PMC7676658; doi:10.1371/journal.pone.0242448)
Supplement: S4 Table — Data is represented as median (IQR). Total NK cell data is presented as a percentage of the lymphocyte gate. Phenotype, functional, survival and proliferation data is presented as a percentage of total NK cells. *ANC associations were calculated from 18 HIV+ participants (ANC was not available for 1 participant). Low NK cell frequencies in some individuals made downstream gating of certain data unreliable. This data has been omitted from the analysis and are indicated as follows: #Medians (IQR) were calculated from 18 HIV+ participants (8 HIV+DARC+); ##Medians (IQR) were calculated from 16 HIV+ participants (8 HIV+DARC- and 8 HIV+DARC+); ###Medians (IQR) were calculated from 19 HIV- (11 HIV-DARC- and 8 HIV-DARC+) and 17 HIV+ (9 HIV+DARC- and 8 HIV+DARC+) participants respectively. Abbreviations: DARC, Duffy Antigen Receptor for Chemokines; IQR, Interquartile range; n, number. (PDF) [file pone.0242448.s006.pdf]

Expression Marker/s	HIV Status			DARC Genotype						ANC Association			
				HIV- (n=20)			HIV+ (n=19)			HIV- (n=20)		HIV+ (n=18)*	
	HIV- (n=20)	HIV+ (n=19)	p value	DARC - (n=12)	DARC+ (n=8)	p value	DARC- (n=10)	DARC + (n=9)	p value	Spearman r	p value	Spearman r	p value
Total NK cells	4.80 (2.90-8.40)	3.4 (1.80-5.70)	0.360	3.28 (1.39-4.90)	8.01 (5.08-13.80)	0.01	2.66 (1.33-9.71)	4.11 (2.43-5.56)	0.40	0.65	0.002	0.31	0.21
CD56 brights	5.57 (3.56-7.00)	3.06 (2.23-5.55)	0.07	6.23 (4.40-10.10)	4.54 (1.92-6.00)	0.08	3.39 (2.23-5.64)	2.94 (1.45-7.67)	0.78	-0.45	0.05	-0.31	0.22
CD56 dims	86.30 (79.80-89.20)	75.50 (62.90-83.50)	0.01	81.70 (78.00-88.60)	88.00 (83.30-90.00)	0.13	74.70 (61.40-87.90)	77.20 (65.80-83.00)	0.90	0.30	0.20	0.07	0.79
CD56 negatives	7.99 (5.78-12.90)	18.10 (9.40-34.20)	0.005	7.44 (5.73-14.80)	8.65 (5.78-12.90)	0.97	18.60 (8.00-36.00)	18.10 (10.10-29.20)	0.97	0.10	0.68	0.02	0.94
CD57+[#]	56.30 (52.10-69.20)	60.40 (51.80-74.80)	0.18	53.80 (46.00-57.20)	68.40 (56.80-75.60)	0.02	64.60 (55.70-74.80)	60.40 (51.60-77.00)	0.90	0.57	0.009	0.01	0.96
KIR+NKG2A-[##]	43.10 (28.20-66.90)	44.70 (25.20-61.00)	0.94	38.90 (35.60-57.80)	48.30 (8.83-78.90)	0.79	44.90 (15.90-73.00)	43.80 (26.80-51.80)	0.89	0.13	0.60	-0.08	0.77
KIR+NKG2A+[##]	6.02 (2.54-16.0)	3.02 (2.12-7.01)	0.05	6.02 (3.11-14.00)	7.60 (2.47-18.50)	0.85	3.13 (2.16-10.90)	2.67 (1.45-3.66)	0.44	-0.01	0.95	-0.47	0.08
KIR-NKG2A+[##]	15.00 (8.37-36.30)	9.55 (5.92-20.00)	0.16	15.00 (9.45-25.30)	16.20 (7.69-58.40)	0.97	9.24 (5.92-23.50)	9.55 (6.61-17.00)	0.96	-0.04	0.87	-0.31	0.25
KIR-NKG2A-[##]	13.30 (7.22-36.30)	36.80 (14.20-58.40)	0.10	27.30 (11.40-47.90)	7.85 (5.44-15.60)	0.06	21.40 (9.90-61.70)	42.70 (18.90-58.40)	0.65	-0.50	0.03	0.35	0.20
HLA-DR+[#]	3.63 (1.94-4.39)	6.38 3.37-10.80)	0.02	3.77 (2.27-6.22)	2.43 (1.43-4.30)	0.42	6.27 (3.37-8.94)	6.53 (2.62-17.10)	0.63	-0.27	0.24	0.11	0.67
PD-1+[#]	0.63 (0.42-1.10)	0.93 (0.62-1.78)	0.09	0.78 (0.48-1.44)	0.42 (0.14-1.00)	0.10	0.76 (0.53-2.18)	1.27 (0.75-2.19)	0.36	-0.39	0.09	0.11	0.68
CD107a+[##]	28.60 (19.90-39.80)	21.20 (14.60-31.20)	0.05	30.30 (20.50-39.70)	28.50 (19.10-40.40)	0.97	20.10 (13.10-30.30)	24.60 (15.50-32.20)	0.51	0.12	0.61	0.20	0.48
TNF-α+[##]	14.10 (7.73-20.50)	9.91 (8.53-20.30)	0.69	15.00 (11.40-22.50)	11.30 (5.86-19.20)	0.46	9.91 (9.08-28.60)	11.10 (6.98-15.30)	0.28	0.007	0.97	0.17	0.55
IFN-γ+[##]	35.50 (32.30-50.00)	37.80 (28.90-46.40)	0.62	39.00 (32.80-50.00)	34.10 (29.50-53.90)	0.67	43.40 (28.50-53.10)	33.50 (28.90-40.80)	0.28	0.13	0.57	0.14	0.61
Ann-PI-, No stim[###]	96.30 (95.00-97.60)	95.30 (91.60-97.40)	0.14	96.20 (93.50-96.80)	96.50 (95.30-98.00)	0.51	94.60 (93.20-96.40)	96.70 (87.50-98.20)	0.54	-0.01	0.96	0.10	0.69
Ann-PI-, K562 + cytokine[###]	87.50 (82.30-90.60)	80.40 (73.90-86.60)	0.02	87.50 (82.30-89.50)	87.90 (79.40-90.80)	0.90	76.80 (73.90-80.50)	86.60 (73.30-91.20)	0.09	-0.06	0.80	0.25	0.32
CFSE low[###]	50.20 (45.00-57.90)	45.30 (35.80-59.10)	0.33	49.90 (45.00-54.20)	51.90 (34.90-64.10)	0.77	43.80 (36.10-63.00)	46.10 (25.80-53.80)	0.67	-0.19	0.43	-0.03	0.91
