# Supplementary material for: The DARC-null trait is associated with moderate modulation of NK cell profiles and unaltered cytolytic T cell profiles in black South Africans
Source: PLoS One. 2020 Nov 19;15(11):e0242448. doi: 10.1371/journal.pone.0242448 (PMC7676658; doi:10.1371/journal.pone.0242448)
Supplement: S5 Table — Data is represented as median (IQR). CD8+ T cell data is presented as a percentage of the lymphocyte gate. Phenotype, functional and proliferation data is presented as a percentage of CD8+ T cells. *ANC associations were calculated from 18 HIV+ participants (ANC was not available for 1 participant). Abbreviations: DARC, Duffy Antigen Receptor for Chemokines; IQR, Interquartile range; n, number. (PDF) [file pone.0242448.s007.pdf]

Expression Marker/s	HIV Status			DARC Genotype						ANC Association			
				HIV- (n=20)			HIV+ (n=19)			HIV- (n=20)		HIV+ (n=18)*	
	HIV- (n=20)	HIV+ (n=19)	p value	DARC - (n=12)	DARC+ (n=8)	p value	DARC- (n=10)	DARC + (n=9)	p value	Spearman r	p value	Spearman r	p value
CD8+ T cells	26.75 (21.30-29.18)	35.41 (26.30-46.87)	0.001	25.60 (19.12-29.60)	26.75 (22.44-28.43)	0.94	38.17 (23.22-50.75)	33.68 (31.43-46.29)	0.97	0.02	0.92	-0.01	0.72
CD57+	24.81 (16.08 - 31.24)	31.27 (18.72 - 38.11)	0.16	24.62 (16.22 - 31.24)	25.56 (16.08 - 33.89)	0.85	29.93 (17.65 - 37.34)	35.31 (24.20 - 38.72)	0.34	0.12	0.61	0.33	0.20
CD38+HLA-DR+	0.40 (0.23 - 0.63)	1.67 (1.21 - 3.04)	<0.0001	0.33 (0.20 - 0.61)	0.49 (0.27 - 1.05)	0.38	1.66 (0.76 - 2.54)	1.85 (1.28 - 4.10)	0.40	0.01	0.96	0.05	0.85
PD-1+	18.21 (12.24 - 24.07)	24.17 15.22 - 32.52)	0.05	15.93 (10.87 - 22.23)	18.21 (15.60 - 25.06)	0.62	15.60 (13.20 - 30.09)	30.49 (19.82 - 37.15)	0.09	0.12	0.63	0.39	0.12
CD107a+, gag	0.00 (0.00 - 0.08)	0.10 (0.00 - 0.48)	0.03	0.00 (0.00 - 0.05)	0.00 (0.00 - 0.12)	0.75	0.05 (0.00 - 0.65)	0.11 (0.02 - 0.40)	0.65	-0.18	0.44	0.32	0.19
CD107a+, gp41	0.00 (0.00 - 0.16)	0.25 (0.18 - 0.45)	0.01	0.05 (0.00-0.18)	0.00 (0.00-0.07)	0.36	0.10 (0.00-0.35)	0.26 (0.15-0.39)	0.28	-0.22	0.34	0.19	0.46
CD107a+, gp120	0.00 (0.00-0.05)	0.02 (0.00-0.70)	0.17	0.00 (0.00-0.09)	0.00 (0.00-0.03)	0.79	0.00 (0.00-0.93)	0.04 (0.01-0.40)	0.42	-0.14	0.55	0.41	0.09
TNF-α+, gag	0.00 (0.00-0.03)	0.01 (0.00-0.11)	0.26	0.00 (0.00-0.03)	0.00 (0.00-0.02)	0.32	0.00 (0.00-0.05)	0.01 (0.00-0.14)	0.83	-0.10	0.66	-0.08	0.76
TNF-α+, gp41	0.00 (0.00-0.04)	0.03 (0.00-0.06)	0.11	0.00 (0.00-0.03)	0.01 (0.00-0.17)	0.26	0.03 (0.00-0.05)	0.03 (0.00-0.36)	0.74	0.14	0.56	-0.14	0.59
TNF-α+, gp120	0.00 (0.00-0.10)	0.00 (0.00-0.07)	0.99	0.01 (0.00-0.11)	0.00 (0.00-0.02)	0.47	0.00 (0.00-0.12)	0.00 (0.00-0.16)	0.57	-0.15	0.54	-0.48	0.04
IFN-ɣ+, gag	0.00 (0.00-0.06)	0.08 (0.00-0.40)	0.02	0.00 (0.00-0.10)	0.00 (0.00-0.03)	0.36	0.07 (0.00-0.40)	0.08 (0.05-0.80)	0.36	-0.10	0.67	-0.01	0.96
IFN-ɣ+, gp41	0.00 (0.00-0.05)	0.21 (0.01-0.41)	0.0036	0.00 (0.00-0.43)	0.00 (0.00-0.00)	0.13	0.18 (0.08-0.59)	0.21 (0.00-0.34)	0.46	-0.67	0.001	0.07	0.78
IFN-ɣ+, gp120	0.05 (0.00-0.14)	0.04 (0.00-0.20)	0.97	0.08 (0.01-0.22)	0.01 (0.00-0.11)	0.19	0.12 (0.00-0.23)	0.02 (0.00-0.06)	0.24	-0.42	0.07	-0.64	0.004
CFSE low	0.32 (0.00-1.83)	2.58 (0.00-11.91)	0.05	0.09 (0.00-3.95)	0.50 (0.00-1.78)	0.97	5.69 (0.00-13.45)	1.41 (0.36-12.43)	0.84	0.17	0.48	-0.14	0.57
